# Supplementary material for: Interpretation of PSMA-PET Among Urologists: A Prospective Multicentric Evaluation
Source: Cancers (Basel). 2025 Jun 24;17(13):2122. doi: 10.3390/cancers17132122 (PMC12249311; doi:10.3390/cancers17132122)
Supplement: Supplementary file 1 [file cancers-17-02122-s001.zip › cancers-3642003-supplementary.pdf]

Supplementary Materials: Interpretation of PSMA-PET Among Urologists: A Prospective Multicentric Evaluation

Guglielmo Mantica, Francesco Chierigo, Francesca Ambrosini, Francesca D'Amico, Greta Celesti, Arianna Ferrari, Fabrizio Gallo, Maurizio Schenone, Andrea Benelli, Carlo Introini, Rosario Leonardi, Alessandro Calarco, Francesco Esperto, Andrea Pacchetti, Rocco Papalia, Giorgio Bozzini, Armando Serao, Valentina Pau, Gianmario Sambuceti, Carlo Terrone, Giuseppe Fornarini and Matteo Bauckneht

Table S1. Correct staging of the twenty cases selected for test administration.

Case number	Tracer Used	Correct Staging
1	18F-PSMA-1007	T3bN0M0
2	18F-PSMA-1007	T3bN0M1c
3	18F-PSMA-1007	T3bN1M1a-c
4	18F-PSMA-1007	T3bN1M1c
5	18F-PSMA-1007	T3bN1M1b
6	18F-PSMA-1007	T2N0M0
7	18F-PSMA-1007	T2N0M0
8	18F-PSMA-1007	T2N1M0
9	18F-PSMA-1007	T2N0M1c
10	18F-PSMA-1007	T2N0M0
11	68Ga-PSMA-11	T3bN1M1a
12	68Ga-PSMA-11	T3bN1M1a
13	68Ga-PSMA-11	T2N0M0
14	68Ga-PSMA-11	T3bN1M1a
15	68Ga-PSMA-11	T2N1M0
16	68Ga-PSMA-11	T3bN1M0
17	68Ga-PSMA-11	T2N1M0
18	68Ga-PSMA-11	T2N0M0
19	68Ga-PSMA-11	T2N1M0
20	68Ga-PSMA-11	T2N0M0
